# Evaluation of the Preventive Effects of Fish Oil and Sunflower Seed Oil on the Pathophysiology of Sepsis in Endotoxemic Rats

**DOI:** 10.3389/fnut.2022.857255

**Published:** 2022-04-06

**Authors:** Yen-Shou Kuo, Mei-Hua Hu, Wei-Hung Chan, Tien-Yu Huang, Yu-Ching Chou, Go-Shine Huang

**Affiliations:** ^1^Division of Thoracic Surgery, Department of Surgery, Tri-Service General Hospital, National Defense Medical Center, Taipei, Taiwan; ^2^Division of Pediatric General Medicine, Chang Gung Memorial Hospital at Linkou, Chang Gung University College of Medicine, Taoyuan, Taiwan; ^3^Graduate Institute of Clinical Medical Sciences, Chang Gung University College of Medicine, Taoyuan, Taiwan; ^4^Department of Anesthesiology, Tri-Service General Hospital, National Defense Medical Center, Taipei, Taiwan; ^5^Division of Gastroenterology, Department of Internal Medicine, Tri-service General Hospital, National Defense Medical Center, Taipei, Taiwan; ^6^School of Public Health, National Defense Medical Center, Taipei, Taiwan

**Keywords:** fish oil, sunflower seed oil, platelet-leukocyte aggregation, P-selectin, CD40L, toll-like receptor 4, multiple organ failure, endotoxemia

## Abstract

Sepsis causes platelet activation, systemic inflammation, organ dysfunction, and mortality. Endotoxins play an important role in the manifestation of the symptoms of septic shock. As fish oil exert well known anti-inflammatory effects and sunflower seed oil exert less anti-inflammatory properties than fish oil, both oils are widely used. We aimed to test the hypothesis that dietary supplementation of these two oils before endotoxemia modulates the consequences of illness. Nine- to ten-week-old male Wistar rats (*N* = 55) were divided into four groups: group A (*N* = 6), control; group B (*N* = 17), saline + lipopolysaccharide (endotoxin); group C (*N* = 17), fish oil + lipopolysaccharide; and group D (*N* = 15), sunflower seed oil + lipopolysaccharide. After 28 days of feeding the designated diet, the rats in all groups were intraperitoneally injected with lipopolysaccharide. After 24 h, survival rate, endotoxemia severity, levels of platelet activation markers, organ function and biochemical variables were evaluated. Platelet-leukocyte aggregation was significantly high in group C (*p* = 0.005), and platelet-monocyte aggregation was significantly high in groups C (*p* = 0.003) and D (*p* = 0.016) than in group B. The survival rate, endotoxemia severity, expression of platelet P-selectin, CD40L, and TLR4, pulmonary function, renal function, liver function, or biochemical variables did not significantly differ among groups B, C, and D. Instead of an anti-inflammatory effect, the dietary supplementation of fish and sunflower seed oils exerted a pro-inflammatory effect, especially *via* platelet-monocyte aggregation, suggesting a rebound effect of the dietary supplementation of the oils. The oils did not affect other inflammatory platelet markers or improve the outcome of endotoxemic rats. However, further studies are required to understand the underlying mechanisms of such effects and to elaborate the clinical significance of these findings.

## Graphical Abstract

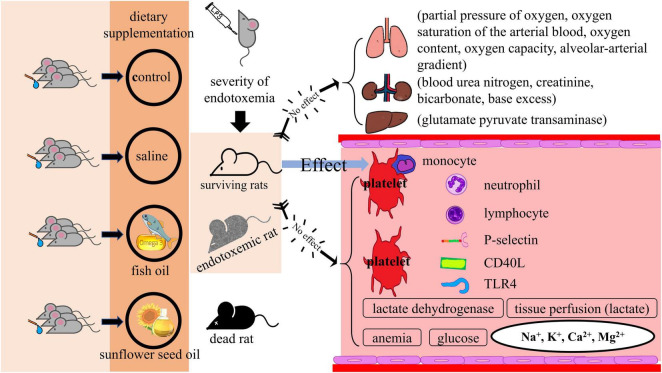

## Introduction

Sepsis is a severe inflammatory disorder as a result of dysregulated host response to infection. During sepsis, underlying circulatory and cellular/metabolic abnormalities can considerably cause multiple organ failure and lead to fatality if not recognized and managed early (1). Platelets play a central role in hemostasis and inflammatory diseases (2), and during sepsis, platelet count and activation are correlated with disease severity and outcome (1, 3). In sepsis, platelets often show high expression of surface-inflammatory markers, including P-selectin, CD40L, and toll-like receptor 4 (TLR4), as well as aggregation of platelet-leukocytes and their subpopulations (4–7).

Fish oil is rich in eicosapentaenoic acid and docosahexaenoic acid and has anti-inflammatory properties (8–12); therefore, it has been recommended as a dietary supplement for the general population to prevent certain pathologies, especially cardiovascular diseases (13). The sunflower seed oil, containing both anti-inflammatory (oleic acid, vitamin E, antioxidants, etc.) and pro-inflammatory (linoleic acid) components (14–20), has lower anti-inflammatory properties than fish oil (21). Although the anti-or pro-inflammatory effect of sunflower seed oil is still a debate, accumulating evidence suggests that it has anti-inflammatory properties (14–20). However, as sunflower seed oil is a widely used cooking oil (14), its effects on sepsis are worth exploring. Thus, there is a need to determine whether dietary supplementation with two anti-inflammatory oils (fish oil and sunflower seed oil) has a preventive effect against endotoxemia, which often progresses to sepsis (22, 23). This study aimed to test the hypothesis that the anti-inflammatory effects of pre-endotoxemia dietary supplementation of fish oil and sunflower seed oil modulate the endotoxemia induced by the lipopolysaccharide (LPS; endotoxin) in rats. To test this hypothesis, we evaluated the survival rate, the severity of endotoxemia, expression levels of platelet-related inflammatory surface markers, organ function and biochemical variables after pre-endotoxemia dietary supplementation of fish oil and sunflower seed oil in an LPS-induced endotoxemic rat model. This study will be an important contribution to the literature because only a few studies have evaluated the preventive effects of dietary supplementation of fish oil and sunflower seed oil on endotoxemia and its related phenotypes.

## Materials and Methods

### Ethics Statement

All procedures were performed in accordance with the Declaration of Helsinki and internationally accepted principles and were approved by the institutional and local committee on the care and use of animals (National Defense Medical Center, Taipei, Taiwan) (Permit number: IACUC-16-135; IACUC-13-190).

### Obtaining Lipopolysaccharide and Dietary Supplements of Fish Oil and Sunflower Seed Oil

Bacterial LPS (*Escherichia coli* serotype 0127:B8), fish oil (from Menhaden fish oil; density: 0.93 g/ml), and sunflower seed oil (from *Helianthus annuus;* density: 0.92 g/ml) were obtained from Sigma-Aldrich, St. Louis, MO, United States (Catalog numbers: L3129, F8020, and S5007, respectively).

### Animals and Experimental Protocol

**FIGURE 1:**
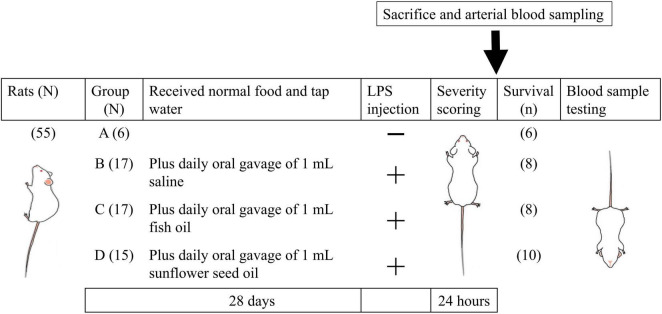
Schematic diagram of grouping and key steps of the study protocol. The rats of all groups were fed normal food and tap water for 28 days, and those in groups B, C, and D were supplemented with daily oral gavage of 1 mL saline, 1 mL fish oil, and 1 mL sunflower seed oil, respectively, for 28 days. On day 29, the rats in all groups, except those in group A, were intraperitoneally injected with *Escherichia coli* LPS (5 mg/kg body weight). LPS, lipopolysaccharide; N, total number of rats; n, number of rats that survived.

Nine- to ten-week-old male Wistar rats, free of specific pathogens (*N* = 55), were purchased from BioLASCO Co., Taipei, Taiwan. The rats were raised and maintained under a 12-/12-h light/dark cycle at a controlled temperature (21°C) in the Laboratory Animal Science Department of the National Defense Medical Center. The scheme of the experimental procedure is shown in Figure 1. The treatments of rats in the four groups with different designated diet were as follows. (1) Group A (*N* = 6), control, rats were fed on normal food and tap water for 28 days, without intraperitoneal injection of *Escherichia coli* LPS; (2) group B (*N* = 17) were fed on normal food and tap water, along with daily oral gavage of 1 mL saline (0.9% sodium chloride solution) for 28 days, followed by intraperitoneal injection of *E. coli* LPS (5 mg/kg); (3) group C (*N* = 17) were fed on normal food and tap water, along with daily oral gavage of 1 mL fish oil for 28 days, followed by intraperitoneal injection of *E. coli* LPS (5 mg/kg); and (4) group D (*N* = 15) were fed on normal food and tap water, along with daily oral gavage of 1 mL sunflower seed oil for 28 days, followed by intraperitoneal injection of *E. coli* LPS (5 mg/kg) (Figure 1).

Twenty-four hours after LPS administration, the survival rate and severity of endotoxemia were measured. The percentage of platelet-leukocyte aggregation and their subpopulations and the platelet surface expression of P-selectin, CD40L, and TLR4 were measured by flow cytometry using the blood of the surviving rats. In addition, we determined pulmonary function (partial pressure of oxygen (PaO_2_), oxygen saturation of the arterial blood (SaO_2_), oxygen content, oxygen capacity, and alveolar-arterial gradient); renal function (blood urea nitrogen (BUN), creatinine, bicarbonate, and base excess); liver function [glutamate pyruvate transaminase (GPT)]; and the levels of hemoglobin, hematocrit, tissue perfusion (lactate), glucose, lactate dehydrogenase (LDH), Na^+^, K^+^, Ca^2+^, and Mg^2+^ (Figure 1).

### Reagents and Antibodies Used for the Detection of Platelet-Leukocyte Aggregation, P-Selectin, CD40L, and TLR4

The leukocyte-common antigens expressed independent of leukocyte activation were identified using anti-CD45-FITC (Beckton, Dickinson, and Co., (BD), Franklin Lakes, NJ, United States) according to the manufacturer’s protocol. The expression of GPIIb/IIIa complex on platelets independent of their activation was determined using anti-CD41a-FITC or anti-CD41a-PE antibody (BD) according to the manufacturer’s protocol. The anti-CD40L-FITC (BD) monoclonal antibody and anti-TLR4-PE monoclonal antibody (BD) were used to determine the expression of CD40L and TLR4, respectively. IgG1κ-FITC and IgG1κ-PE antibodies (BD) were used to assess non-specific binding. The platelet wash buffer and HEPES-buffered Tyrode’s solution were prepared in our laboratory (24, 25).

### Evaluation of Severity of Endotoxemia

After 28 days of treatment, the mean body weight of the rats was 461.45 g (Table 1). Before their sacrifice at prearranged time points, the rats were scored as mildly, moderately, or severely affected by endotoxemia. The scoring was based on appearance, alertness, and blood pressure (26). The rats were to present at least two characteristics from the appearance and alertness categories (Supplementary Table 1) in order for a score to be provided.

**TABLE 1:** Mean body weight of rats of four groups.

Groups	Weight (g)
Control	478.33 ± 24.83
Saline + LPS	479.41 ± 48.54
Fish oil + LPS	441.18 ± 34.07
Sunflower seed oil + LPS	457.33 ± 23.14
Total	461.45 ± 38.8

*Data are expressed as mean ± SD.*

### Blood Sampling

After 24 h of the injection of LPS, blood samples (8–10 ml) from rats in the four groups were obtained using a catheter placed in the right carotid artery under deep terminal anesthesia. All samples were anticoagulated using a 1:9 volume of 3.8% sodium citrate solution.

### Sacrifice of Rats

After blood collection, the rats were euthanized with a lethal dose of pentobarbital.

### Detection of Platelet-Leukocyte Aggregation in Whole Blood

The platelet-leukocyte aggregation in whole blood was detected following the method described in our previous study (25). Briefly, whole-blood samples were mixed with FACS™ lysing solution (BD) and incubated for 20 min for the red blood cells (RBCs) to lyse, followed by 6 min centrifugation at 400 × *g*. After discarding the supernatant, the pellet was resuspended in phosphate-buffered saline (PBS). The suspension was centrifuged at 400 × *g* for 6 min, and platelet-leukocyte aggregate was obtained by discarding the supernatant and resuspending the pellet in PBS. The samples were stained with anti-CD45-FITC and anti-CD41a-PE monoclonal antibody, to identify leukocytes and platelets, respectively, and incubated for 20 min in the dark to determine platelet-leukocyte aggregation. Platelet-coupled and platelet-free leukocytes were determined using two-color labeling (CD41a-PE vs. CD45-FITC). The percentage of platelet-coupled leukocytes was calculated in the leukocyte population. The percentages of platelet-neutrophil, platelet-monocyte, and platelet-lymphocyte aggregates were measured, respectively. Finally, 10,000 leukocytes were counted in each sample.

### Preparation of Platelet-Rich Plasma and Washed Platelets

Platelet-rich plasma was obtained by centrifuging the whole blood at 200 × *g* for 10 min. Next, platelet-rich plasma was centrifuged at 2,000 × *g* for 10 min. The pellet was washed by resuspending in platelet washing buffer, followed by 10 min centrifugation at 2,000 × *g*. The supernatant was discarded, and the washed platelets were resuspended in HEPES-Tyrode buffer. The suspension was diluted to a final platelet count of 150,000–450,000 platelets/μl.

### Detection of Platelet P-Selectin (CD62P) Expression in Platelet-Rich Plasma

To determine the expression of P-selectin, the platelet-rich plasma was incubated in the dark with anti-CD41a-FITC and anti-CD62P-PE monoclonal antibodies at room temperature (∼22–28°C) for 20 min. The platelets were individually identified by measuring the side scatter and anti-CD41a-FITC immunofluorescence on a logarithmic scale dot plot. For background control, the platelets were incubated with FITC-labeled mouse IgG1κ and PE-labeled mouse IgG1κ. The results are expressed as the average fluorescence intensity of CD62P-PE, and 10,000 platelet readings were recorded for each sample.

### Detection of CD40L and TLR4 Expression in Washed Platelets

To determine the expression of CD40L and TLR4, the samples were stained with anti-CD41a-PE and anti-CD40L-FITC, and anti-CD41a-PE and anti-TLR4-FITC monoclonal antibodies, respectively, and incubated at 22–28°C for 20 min in the dark. Platelets were individually identified by measuring the side scatter and anti-CD41a-PE immunofluorescence in a logarithmic scale dot plot. For background control, the platelets were incubated with FITC-labeled mouse IgG1κ and PE-labeled mouse IgG1κ. The results are expressed as the average fluorescence intensity of TLR4-FITC, and 10,000 platelet readings were recorded for each sample.

### Flow Cytometry

For cytometric analysis, a FACSCalibur™ flow cytometer (BD), a standard two-color filter configuration, and CellQuest cell analysis software (BD) were used.

### Arterial Blood Gas Analysis, Organ Function/Injury Quantification, and Biochemical Data Collection

Whole-blood gas analysis was performed using an arterial blood gas analyzer (AVL OPTI Critical Care Analyzer; AVL Scientific Corp., Roswell, GA, United States). Whole blood was centrifuged at 16,000 × *g* for 2 min to obtain serum for measuring biochemical variables. The biochemical variables were analyzed using Fuji DRI-CHEM 3030 (Fuji Photo Film, Tokyo, Japan). A combination of both arterial blood gas and biochemical variables analyzers can estimate the parameters for arterial blood gas, biochemical data, and organ function/injury parameters, such as measurement of pulmonary function (PaO_2_, SaO_2_, oxygen content, oxygen capacity, and alveolar-arterial gradient); renal function (BUN, creatinine, bicarbonate, and base excess); liver function (GPT); and levels of hemoglobin, hematocrit, tissue perfusion (lactate), glucose, LDH, Na^+^, K^+^, Ca^2+^, and Mg^2+^.

### Statistical Analyses

The primary outcome measures were survival rate and severity of endotoxemia. The secondary outcome measures were the percentage of platelet-leukocyte aggregation and platelet surface expression levels of P-selectin, CD40L, and TLR4. Continuous measurements are presented as mean ± standard deviation (SD), and categorical variables are reported as number and percentage (%). The survival rate and severity of endotoxemia were measured using the chi-square test. Results from platelet surface expression levels of P-selectin, CD40L, and TLR4; arterial blood gas parameters; biochemical variables; and organ function were analyzed using Kruskal–Wallis test followed by Mann–Whitney *U* test. SPSS software (Version 20; SPSS, Inc., Chicago, IL, United States) was used for all analyses. Statistical significance was set at *p* < 0.05. All data are presented as mean ± SD. In addition, with the use of G*Power 3.1.3 software, a power analysis was performed using the chi-square test among group B, C, and D. After ordering α = 0.05, *n* = 17 in group B and C and *n* = 15 in group D, we calculated the power (1-β) = (5–62.1%), (14.9–70.6%), (5–18.5%), and (5–14.3%) for survival rate, mild sepsis rate, moderate sepsis rate, and severe sepsis rate, respectively, among three group (group B vs. C, group B vs. D, and group C vs. D).

## Results

### Effects of Dietary Supplementation of Fish Oil and Sunflower Seed Oil on the Survival Rate and Severity of Endotoxemia

**FIGURE 2:**
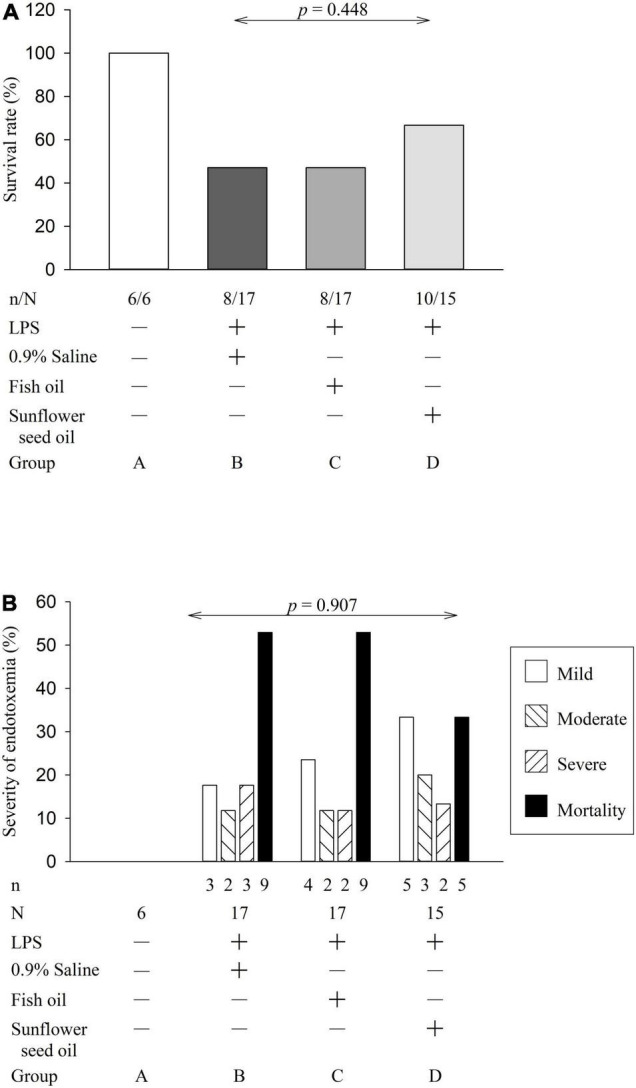
Effects of dietary supplementation of fish oil and sunflower seed oil on survival rate **(A)** and severity of endotoxemia **(B)**. Data are expressed as a percentage (%). LPS, lipopolysaccharide; N, total number of rats of each group; n, number of rats that survived.

The clinical manifestations of the surviving rats with endotoxemia, progressing to sepsis, included a hunched appearance, piloerection, bloating, and a loss of interest in their environment, the severity of which allowed clinical grading of the rats into mild, moderate, and severe illness groups. However, we did not observe a statistically significant difference in the survival rate (*p* = 0.448) or severity of endotoxemia (*p* = 0.907) among groups B, C, and D (Figure 2), indicating no effect of dietary supplementation of fish oil and sunflower seed oil on the alleviation of endotoxemia.

### Effects of Dietary Supplementation of Fish Oil and Sunflower Seed Oil on Platelet-Leukocyte Aggregation and Its Subpopulations in Endotoxemic Rats

**FIGURE 3:**
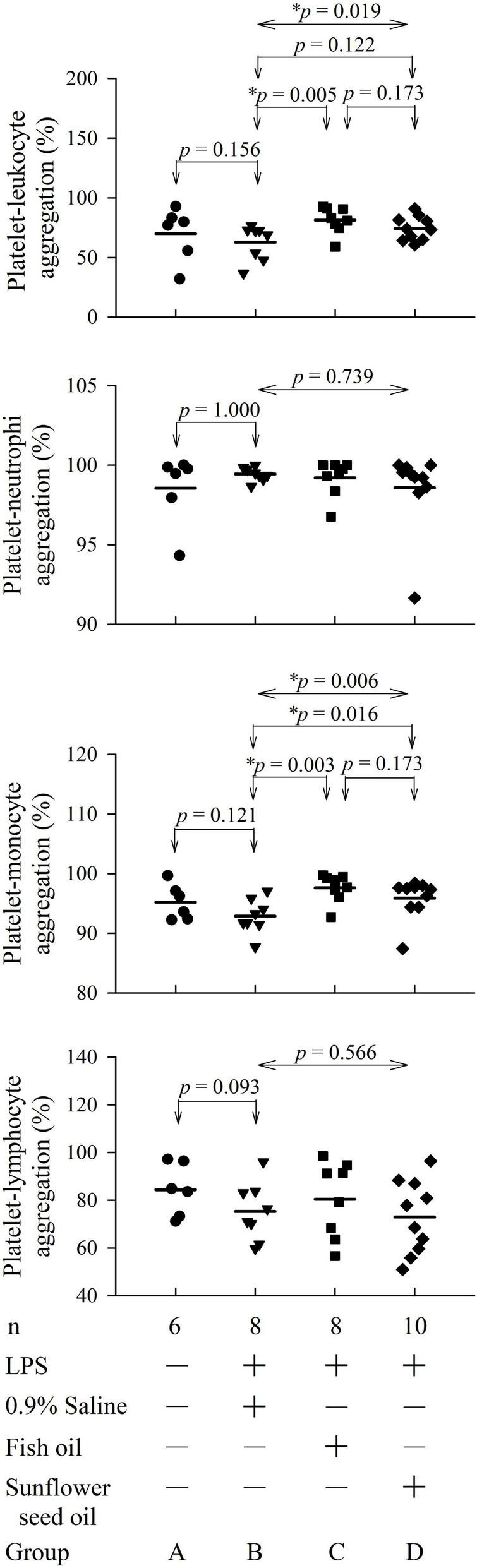
Effects of dietary supplementation of fish oil and sunflower seed oil on platelet-leukocyte aggregation and its subpopulations in endotoxemic rats. Data are expressed as mean ± SD. LPS, lipopolysaccharide; n, number of rats that survived. *p < 0.05.

The flow cytometry analyses showing platelet-leukocyte aggregation are displayed in Supplementary Figure 1. Group C (*p* = 0.005), but not group D (*p* = 0.122), showed significant elevation in platelet-leukocyte aggregation than group B. In the platelet-leukocyte aggregation subpopulations, only platelet-monocyte aggregation was statistically different in group C (*p* = 0.003) and group D (*p* = 0.016) compared with that in group B. However, platelet-neutrophil (*p* = 0.739) and platelet-lymphocyte (*p* = 0.566) aggregation did not present significant differences among the three groups (Figure 3 and Supplementary Table 2).

### Effects of Dietary Supplementation of Fish Oil and Sunflower Seed Oil on Platelet P-Selectin, CD40L, and TLR4 Expression in Endotoxemic Rats

**FIGURE 4:**
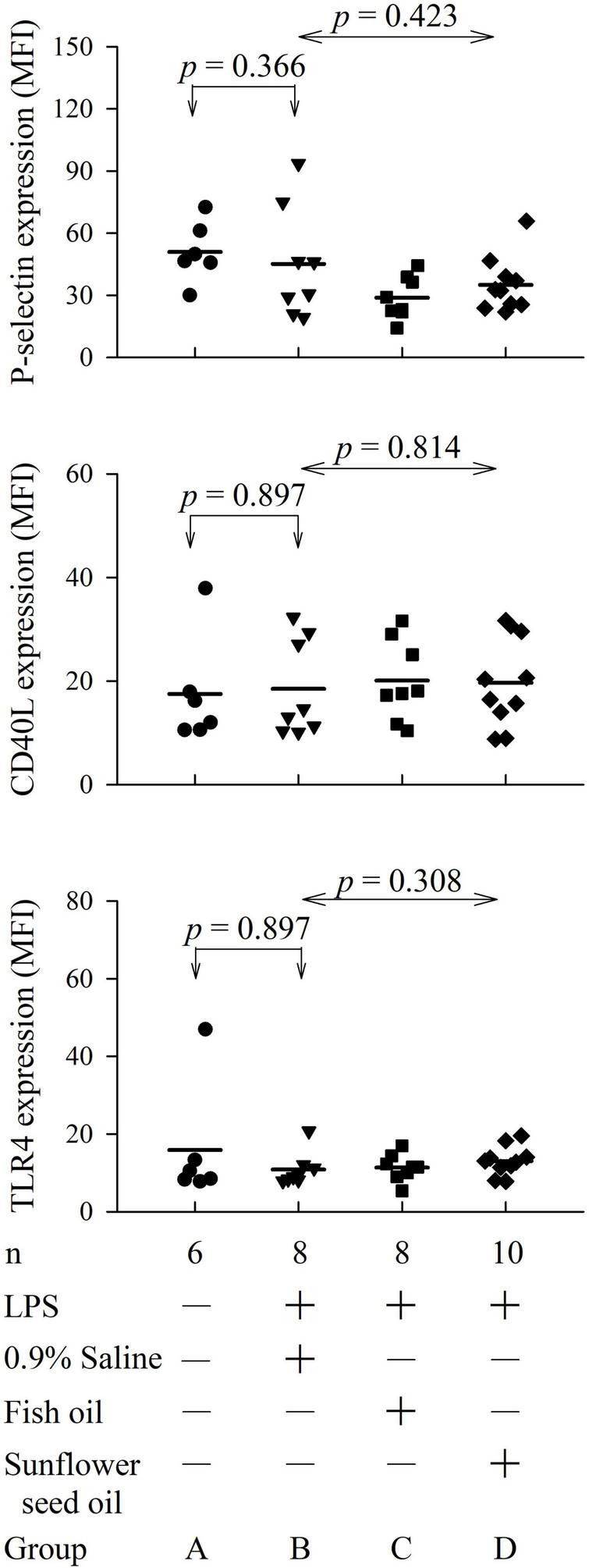
Effects of dietary supplementation of fish oil and sunflower seed oil on platelet P-selectin, CD40L, and TLR4 expression in endotoxemic rats. Data are expressed as mean ± SD. MFI, mean fluorescence intensity; LPS, lipopolysaccharide; n, number of rats that survived.

The flow cytometry analyses for platelet P-selectin expression, CD40L expression, and TLR4 expression are displayed in Supplementary Figures 2–4. The four groups showed no significant differences among groups B, C, and D in the expression of platelet P-selectin (*p* = 0.423), CD40L (*p* = 0.814), or TLR4 (*p* = 0.308) (Figure 4 and Supplementary Table 3).

### Effects of Dietary Supplementation of Fish Oil and Sunflower Seed Oil on Arterial Blood Gas Parameters, Biochemical Variables, and Organ Function and Injury in Endotoxemic Rats

**FIGURE 5:**
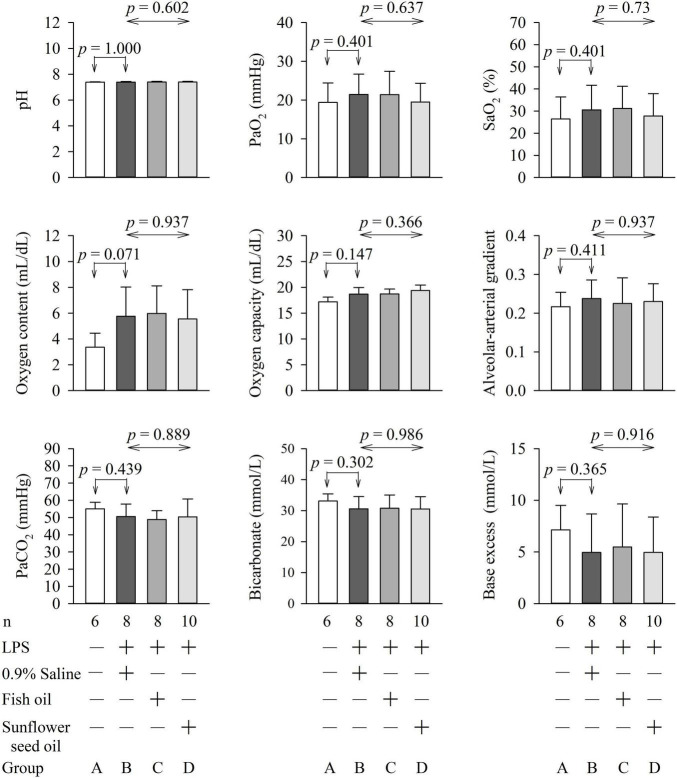
Effects of dietary supplementation of fish oil and sunflower seed oil on pH, PaO_2_, SaO_2_, oxygen content, oxygen capacity, alveolar-arterial gradient, PaCO_2_, and Bicarbonate and base excess. Data are expressed as mean ± SD. LPS, lipopolysaccharide; n, number of rats that survived.

**FIGURE 6:**
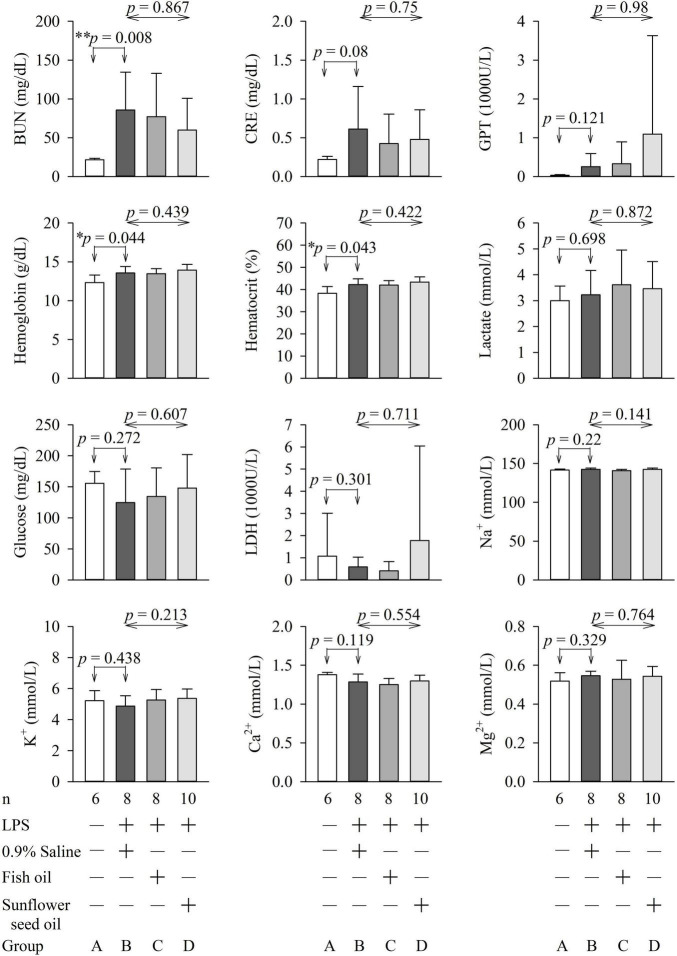
Effects of dietary supplementation of fish oil and sunflower seed oil on the levels of BUN, creatinine, GPT, hemoglobin, hematocrit, lactate, glucose, LDH, Na^+^, K^+^, Ca^2+^, and Mg^2+^). Data are expressed as mean ± SD. BUN, blood urea nitrogen; GPT, glutamate pyruvate transaminase; LDH, lactate dehydrogenase; LPS, lipopolysaccharide; n, number of rats that survived.

The parameters of arterial blood gas, biochemical variables, and organ function and injury did not show any significant differences among groups B, C, and D (Figures 5, 6 and Supplementary Tables 4, 5).

## Discussion

We found that fish oil and sunflower seed oil could induce platelet-leukocyte aggregation (group C) and platelet-monocyte aggregation (groups C and D), indicating increased inflammation during endotoxemia. However, the survival rate, the severity of endotoxemia, percentage of platelet-neutrophil and platelet-lymphocyte aggregation, platelet P-selectin, CD40L, TLR4 expression, organ injury, tissue perfusion, and biochemical variables were not significantly different among groups B, C, and D.

Platelet-leukocyte aggregation is a considerable pathological feature of sepsis (4). Although fish oil is known to exhibit anti-inflammatory activity (8, 9, 13), we showed that pre-endotoxemia dietary supplementation of fish oil exerted a pro-inflammatory effect by increasing platelet-leukocyte aggregation in endotoxemic rats. In contrast, the dietary supplementation of sunflower seed oil showed no anti-inflammatory effect or effects on the platelet-leukocyte aggregation-related pro-inflammatory responses. Platelet-leukocyte aggregation mainly occurs through the binding of the P-selectin on platelets to P-selectin glycoprotein ligand-1 expressed on leukocytes, which is then stabilized through the binding of glycoprotein Ib and ICAM-2 on platelets to Mac-1 and lymphocyte function-associated antigen-1 on leukocytes, respectively (25). Platelet-leukocyte aggregation also depends on leukocyte activation. However, leukocyte activation has not been evaluated in this study.

The platelet-leukocyte aggregation has three major subpopulations—platelet-neutrophil, platelet-monocyte, and platelet-lymphocyte aggregations. In this study, only the platelet-monocyte aggregation increased significantly in endotoxemic rats upon pre-endotoxemia dietary supplementation of both fish oil and sunflower seed oil (Figure 3). Reportedly, platelet-monocyte aggregates increase mortality risk, especially in aged patients with sepsis and septic shock (27). The level of platelet-monocyte aggregates has been shown to increase in a murine sepsis model, suggesting their pro-inflammatory roles in sepsis (28, 29). Further, this aggregation may help identify therapeutic targets that can positively affect the outcome of sepsis (30). Taken together, platelet-monocyte aggregation is considered an important prognostic indicator of sepsis. The findings of the present study demonstrate that regardless of dietary supplementation of fish oil and sunflower seed oil, platelet-monocyte aggregation-related inflammation may elevate pre-existing sepsis inflammatory status during sepsis. Therefore, we do not suggest pre-sepsis dietary supplementation of fish oil and sunflower seed oil for routine enteral intake due to our unexpected results in endotoxemic rats. These suggestion are in accordance with the guidelines of the American Society for Parenteral and Enteral Nutrition (A.S.P.E.N.) for the provision of nutrition support therapy in critically ill adult patients, which suggest that immune-modulating formulas (including omega-3 fish oils) often showed negative results in such patients and thus cannot be used routinely in patients with sepsis (9, 31). However, the results of several meta-analyses are not consistent—some meta-analyses have reported that Omega-3 fatty acid supplementation is associated with favorable outcomes in such patients (12). These differences could be attributed to the inclusion and exclusion criteria, clinical settings, the number of patients studied, stages of sepsis evaluated, etc. Therefore, to ascertain the effects of the supplementation of fish or sunflower seed oil, the results of our study should be evaluated clinically using a large number of patients with sepsis at different stages.

We further investigated platelet-neutrophil and platelet-lymphocyte aggregations because they can induce inflammatory responses in endotoxemic rats (Figure 3). Wang et al. suggested that targeting platelet-neutrophil interactions could be a new strategy for sepsis treatment (4). Furthermore, platelet-lymphocyte aggregation is an important regulatory mechanism in thrombosis, inflammation, immunological reactions, and atherosclerosis (32). However, our results indicated that platelet-monocyte aggregation is a more sensitive inflammatory marker in endotoxemia than platelet-neutrophil and platelet-lymphocyte aggregations. This is consistent with the results of Michelson et al. in patients with acute myocardial infarction (33).

Our results showed that supplementation with fish oil or sunflower oil, despite their anti-inflammatory properties, not only aggravated the inflammatory conditions after endotoxemia but also increased the pro-inflammatory status by inducing platelet-leukocyte aggregation. This could be attributed to the rebound effect of fish oil or sunflower oil that might have suppressed the anti-inflammatory effects of these oils, or non-responsiveness of the rats after LPS injection due to the aggravated negative symptoms (34). The increased platelet-monocyte aggregation in group C than in group D could be explained by the stronger anti-inflammatory effect of fish oil than sunflower seed oil; therefore, leading to more pronounced rebound effect, and as a result a higher level of platelet-monocyte aggregation in the fish oil group than sunflower seed oil group. The rebound effects of drugs are well documented in several studies (34–36). For instance, atorvastatin withdrawal led to a rebound increase in inflammatory mediators, as a result, patients with myocardial infarction might have adverse rebound effect after cessation of statin therapy (36).

To comprehensively analyze the inflammatory effects of the dietary supplements, we investigated other clinically important inflammatory platelet markers. Individuals with sepsis often show a high surface expression of platelet P-selectin, CD40L, and TLR4. For example, the inflammatory marker P-selectin can predict the duration of hospital stay and 30-day survival in patients with sepsis (5). CD40L signaling regulates platelet activation and endothelial cell damage during sepsis (6). Furthermore, sepsis activates the TLR4 pathway to promote macrophage infiltration, thereby impeding neuromuscular function (7). Hence, the combination of TLR4 and CD40L could predict the early diagnosis and severity of sepsis (37). However, this study finding implied that dietary supplementation of fish oil and sunflower seed oil exerts no significant effect on the survival rate, the severity of endotoxemia, and inflammatory status *via* the P-selectin, CD40L, and TLR4 pathways (Figure 4).

The PaO_2_ and SaO_2_ were low, whereas the PaCO_2_ was high in the four groups. This could possibly be due to hypoventilation arising from the deep terminal anesthesia administered to the rats before collection of blood samples. Furthermore, endotoxemia with illness also compromises the respiratory drive of rats. Pulmonary dysfunction, due to endotoxemia with acute respiratory distress syndrome, was less likely because the rats in group A (without endotoxemia) also showed the same pattern.

Blood glucose levels are important for sepsis, as hyperglycemia (>180 mg/dL), hypoglycemia, and increased glycemic variability are associated with high mortality in critically ill patients (23). The lactate level guides resuscitation in sepsis and is related to the prognosis of patients with sepsis (38). LDH is a marker of the degree of tissue injury, and increased plasma LDH levels are commonly observed in patients with severe sepsis (23). Hence, these parameters were assessed in this study. We found that pulmonary function (PaO_2_, SaO_2_, oxygen content, oxygen capacity, and alveolar-arterial gradient); renal function (BUN, creatinine, bicarbonate, and base excess); liver function (GPT); and levels of hemoglobin, hematocrit, tissue perfusion (lactate), glucose, LDH, Na^+^, K^+^, Ca^2+^, and Mg^2+^ were not significantly different among groups B, C, and D (Figures 5, 6). This implies that fish oil- or sunflower seed oil-based dietary supplements have no preventive or aggravative effects on the consequence of endotoxemia.

Sepsis is a biphasic disease, and its initial phase is characterized by massive inflammation followed by immunosuppression. However, most deaths occur during the immunosuppression stage of this biphasic disease (39). During the treatment of sepsis, the two stages are not easy to differentiate. Therefore, while the management of sepsis is related to inflammation, anti-inflammation is not always effective. Sepsis-related inflammation is a necessary evil (39). Hence, a homeostatic balance between these competing events is necessary for recovery. This two-stage phenomenon might explain why our preventive treatment with fish and sunflower seed oils did not affect the survival rate, the severity of endotoxemia, expression of inflammatory markers, or organ function parameters.

In this study, fish oil was chosen based on its composition with well-known nutritional and pharmaceutical properties (8–10). These include anti-inflammatory properties due to the presence of eicosapentaenoic acid and docosahexaenoic acid, resulting in a higher proportion of eicosapentaenoic acid and docosahexaenoic acid in the cell membrane and a lower proportion of arachidonic acid, thereby inhibiting the synthesis of inflammatory eicosanoids and reducing the production of inflammatory cytokines, including TNF-α, IL-6, and IL-8 (8–11). The sunflower seed oil was chosen based on its composition and the fact that it is recognized as a nutritional and healthy food (14–18); however, it is still not used as a dietary supplement like fish oil. Sunflower is one of the main crops used for edible oil production in several countries. Sunflower seed oil comprises both anti-inflammatory and pro-inflammatory constituents. One pro-inflammatory constituent is linoleic acid, a polyunsaturated fat present in considerably high amounts (55%–70%). It is considered that increasing the dietary intake of the omega-6 fatty acid (arachidonic acid) or its precursor linoleic acid can enhance inflammation. However, studies based on healthy human adults have shown that increased intake of arachidonic acid or linoleic acid does not increase the levels of inflammatory markers (20). Several observations support the anti-inflammatory properties of sunflower seed oil. First, sunflower seed oil contains a considerable amount of oleic acid (a mono-saturated fat, 20–25%) (14) that is known to exert anti-inflammatory effects (19). Second, sunflower seed oil contains magnesium and vitamin E, well-known antioxidants with anti-inflammatory properties. Third, the sunflower seed oil is rich in enzymes, phenolic compounds, carotenoids, L-ascorbic acid, and peptides that exert anti-inflammatory effects (14). Besides, linoleic acid-induced inflammation may be reversed by the anti-inflammatory effect of oleic acid, vitamin E, antioxidants, and other anti-inflammatory components. Therefore, collectively, available evidence suggests that sunflower seed oil is more likely to have overall anti-inflammatory properties (14–20).

Despite not finding any specific effect on the survival rate or severity of endotoxemia upon dietary supplementation with fish oil and sunflower seed oil, both are thought to be good for the general health of the patients. Fish oil is beneficial in conditions other than sepsis, such as traumatic brain injury (9). Furthermore, an immune-modulating formula (containing both arginine and fish oil) in the surgical intensive care unit for postoperative patients who require enteral nutrition therapy is beneficial (9). The biological effects of sunflower seed oil include antioxidant, anti-inflammatory, antidiabetic, antimicrobial, and antihypertensive effects. The health benefits of sunflower seed oil include blood pressure and diabetes control, and skin protection (14).

Our study diet is similar to the Mediterranean diet, containing fish oil (40). Many doctors and dietitians recommend a Mediterranean diet to maintain good health and prevent diseases. Even fish oil-based dietary supplements (40) are often recommended to be taken daily (13). A study showed some evidence of the possible effect of the Mediterranean diet on general health (40). However, our results for pre-endotoxemia dietary supplementation of fish oil and sunflower seed oil, consistent with the guidelines for nutrition support therapy in critically ill adult patients, showed no benefit for sepsis (9, 31). Hence, standard enteral nutrition formula for patients with sepsis might be considered (8, 9, 31). Furthermore, a Mediterranean-style diet includes a variety of foods, including high consumption of fish, fruit, vegetables, legumes, and cereals, and low consumption of meat, dairy products, fat, and alcohol. Fish oil or sunflower seed oil alone may not be sufficient to improve the outcome of endotoxemia with illness.

This study has several strengths. We employed a comprehensive search strategy as a part of a multi-faceted assessment to examine preventive interventions of the anti-inflammatory effects of oils in treating endotoxemia. The outcome of this study is meaningful in exploring the clinical significance of the dietary supplementation of these oils while considering the rebound effect. The study unraveled that neither of the oils studied showed preventive effects against endotoxemia; therefore, it can contribute to the scientific literature by forcing us to critically re-evaluate a relatively unexplored aspect of nutrition.

Our study had the following limitations. First, although it is better to prevent sepsis from occurring than cure it, prevention is difficult to achieve in humans. Studying non-human animals is a useful way to further our understanding, but it may not provide suitable predictors for humans. Therefore, a retrospective study involving patients with sepsis who have taken dietary supplements of fish oil or use sunflower seed oil for cooking may be warranted (23, 38, 41). Second, for the quantitative monitoring for the overview of endotoxemia development, inflammatory factors such as TNF-alpha, IL-6, IL-8, IL-4, and some leukocyte antigens, such as macrophage-1 antigen (25) expression at different time points such as 0, 6, 10 and 24 h after LPS injection could be measured. However, we shall not reach the consequences of our aim to measured survival rate and severity of endotoxemia. Third, in this study, some rats died within 24 h after LPS intraperitoneal injection in groups B, C and D. Therefore, blood samples were not obtained from the dead rats. This may contribute to some degree of bias. Reducing the LPS dose to increase the survival rate or inclusion of more rats in the test groups should be considered. Nevertheless, the three Rs (Replacement, Reduction and Refinement) principles should be respected for the ethical use of animals in product testing and scientific research. Fourth, the endotoxemic rats developed even severe illness of endotoxemia but did not receive appropriate treatments, including sets of hour-1 bundle (23, 38). Both sepsis and septic shock are medical emergencies requiring rapid diagnosis and immediate intervention. Thus, a prompt treatment, especially the hour-1 bundle (23, 38), may change the disease outcome of endotoxemic rats (23). The important relationship between the bundles and survival was confirmed (23, 41). However, our protocol is similar to the lack of medical resources or in battlefields.

## Conclusion

This study is novel because, to our knowledge, only a few studies have evaluated the preventive effects of the dietary supplements of fish oil and sunflower seed oil on the severity of endotoxemia, survival rate, levels of inflammatory platelet markers, and organ dysfunction. We demonstrated that pre-endotoxemia dietary supplements of fish oil and sunflower seed oil are not sufficient as standalone treatments for endotoxemia. Sepsis therapy is hampered because hyper-inflammatory and hypo-inflammatory phases alternate, requiring an adaptive approach. Fish and sunflower seed oil supplementation showed no superiority over normal feeding in endotoxemia. Furthermore, consistent with the A.S.P.E.N. guidelines for the provision of nutrition support therapy in critically ill adult patients, the use of immune-modulating formulas, including fish oil, did not show outcome benefits over standard enteral nutrition formulas in a medical intensive care unit setting. Therefore, the study findings suggest that the significance of fish oil and sunflower seed oil in the intensive care unit setting should not be overstated, considering our findings from administration during and before endotoxemia. Our results imply dietary supplements should be developed conservatively and cautiously to prevent the progress of endotoxemia to sepsis and mortality. However, animal research is not sufficient to fully understand their effect on humans; hence, further studies are necessary.

## Data Availability Statement

The original contributions presented in the study are included in the article/Supplementary Material, further inquiries can be directed to the corresponding author/s.

## Ethics Statement

The animal study was reviewed and approved by National Defense Medical Center, Taipei, Taiwan; Permit number: IACUC-16-135; IACUC-13-190.

## Author Contributions

Y-SK, M-HH, T-YH, W-HC, and G-SH conceived and designed the study and conducted data screening and extraction. Y-SK, M-HH, T-YH, Y-CC, and G-SH conducted data analysis. G-SH drafted the manuscript with critical inputs from all co-authors. All authors approved the final manuscript.

## Conflict of Interest

The authors declare that the research was conducted in the absence of any commercial or financial relationships that could be construed as a potential conflict of interest.

## Publisher’s Note

All claims expressed in this article are solely those of the authors and do not necessarily represent those of their affiliated organizations, or those of the publisher, the editors and the reviewers. Any product that may be evaluated in this article, or claim that may be made by its manufacturer, is not guaranteed or endorsed by the publisher.
