# Supplementary material for: Evaluation of the Preventive Effects of Fish Oil and Sunflower Seed Oil on the Pathophysiology of Sepsis in Endotoxemic Rats
Source: Front Nutr. 2022 Apr 6;9:857255. doi: 10.3389/fnut.2022.857255 (PMC9026188; doi:10.3389/fnut.2022.857255)
Supplement: Supplementary file 1 [file Data_Sheet_1.PDF]

Supplementary Material

1 Supplementary Figures and Tables

1.1 Supplementary Tables

	Mild	Moderate	Severe
Appearance	Hunched<br>Piloerection<br><br>No bloating	Hunched<br>Marked piloerection<br>Bloated abdomen<br>Sunken eyes	Marked piloerection<br>Marked bloated abdomen<br>Conjunctival injection
Alertness	Alert<br><br>Occasional interest in environment<br>Moves freely	Depressed level of alertness<br><br>Little interest in environment<br>Moves with difficulty	Markedly (or absent) depressed level of alertness<br>No interest in environment<br>No movement
Mean blood pressure	>90 mmHg	75-90 mmHg	<75 mmHg

Table S1. Severity scoring system. (1)

Groups / Variables		A	B	C	D	P^1	P^2
Platelet-leukocyte aggregation (%)	Median (IQR)	78.39 (35.75)	70.63 (23.62)	82.13 (15.43)	73.68 (17.63)	*0.156	0.019
	Mean ± SD	70.06 ± 22.25	62.77 ± 14.64	81.31 ± 11.08	74.35 ± 10.03		
Platelet-neutrophil aggregation (%)	Median (IQR)	99.63 (2.86)	99.44 (0.68)	99.64 (1.39)	99.35 (1.34)	1.000	0.739
	Mean ±	98.57 ±	99.46 ±	99.22 ±	98.59 ±		

	SD	2.21	0.44	1.13	2.5		
Platelet-monocyte aggregation	Median (IQR)	94.93 (5.38)	92.54 (3.87)	98.33 (3.03)	97.42 (3.38)	0.121	**0.006
	Mean ± SD	95.21 ± 2.96	92.89 ± 2.9	97.65 ± 2.35	95.92 ± 3.3		
Platelet-lymphocyte aggregation (%)	Median (IQR)	84.15 (23.82)	73.74 (19.88)	85.19 (29.03)	73.18 (28.58)	0.093	0.566
	Mean ± SD	84.35 ± 11	75.24 ± 12.17	80.44 ± 15.87	72.92 ± 15.37		

Table S2. Effects of dietary supplementation of fish oil and sunflower seed oil on platelet-leukocyte aggregation and its subpopulations in endotoxemic rats. LPS = lipopolysaccharide. n = number of rats that survived. *$p < 0.05$.

[1]Group B compared with that in group A.

[2]Statistical comparison among groups B, C, and D.

Groups / Variables		A	B	C	D	P^1	P^2
P-selectin expression (MFI)	Median (IQR)	48.19 (22.19)	38.42 (44.74)	26.24 (16.09)	35.29 (15.84)	0.366	0.423
	Mean ± SD	51 ± 14.52	45.21 ± 26.61	28.9 ± 10.23	35.14 ± 13.26		
CD40L expression (MFI)	Median (IQR)	14.13 (12.31)	13.83 (18.18)	17.87 (14.99)	18.44 (17.12)	0.897	0.814
	Mean ± SD	17.55 ± 10.42	18.55 ± 9.37	20.13 ± 7.75	19.71 ± 8.54		
TLR4 expression (MFI)	Median (IQR)	9.48 (13.58)	9.23 (3.62)	11.5 (4.62)	12.88 (4.55)	0.897	0.308
	Mean ± SD	15.87 ± 15.36	10.86 ± 4.26	11.36 ± 3.48	13.04 ± 3.77		

Table S3. Effects of dietary supplementation of fish oil and sunflower seed oil on platelet P-selectin, CD40L, and TLR4 expression in endotoxemic rats. MFI = mean fluorescence intensity, LPS = lipopolysaccharide, n = number of rats that survived.

[1]Group B compared with that in group A.

[2]Statistical comparison among groups B, C, and D.

Groups / Variables		A	B	C	D	P^1	P^2
pH	Median (IQR)	7.39 (0.08)	7.39 (0.07)	7.42 (0.06)	7.39 (0.05)	1.000	0.602
	Mean ± SD	7.38 ± 0.04	7.39 ± 0.06	7.4 ± 0.05	7.39 ± 0.06		
PaO_2 (mmHg)	Median (IQR)	18.35 (10.1)	20.9 (9.6)	19.15 (11.25)	17.85 (8.75)	0.401	0.637
	Mean ± SD	19.38 ± 5.52	21.45 ± 5.61	21.39 ± 6.45	19.48 ± 5.14		
SaO_2 (%)	Median (IQR)	25.25 (21.8)	29.2 (14.4)	28.6 (19.95)	26.65 (18.95)	0.401	0.73
	Mean ± SD	26.48 ± 10.85	30.56 ± 11.94	31.24 ± 10.69	27.8 ± 10.68		
Oxygen content (mL/dL)	Median (IQR)	2.7 (n<4)	5.45 (4.53)	5.35 (4.23)	5.25 (3.98)	0.071	0.937
	Mean ± SD	3.37 ± 1.33	5.77 ± 2.47	5.98 ± 2.29	5.55 ± 2.4		
Oxygen capacity (mL/dL)	Median (IQR)	17.4 (n<4)	18.3 (2.63)	18.6 (1.58)	19.35 (1.68)	0.147	0.366
	Mean ± SD	17.2 ± 1.11	18.68 ± 1.41	18.74 ± 0.99	19.39 ± 1.11		
Alveolar-arterial gradient	Median (IQR)	0.2 (0.03)	0.2 (0.1)	0.2 (0.1)	0.2 (0.1)	0.411	0.937
	Mean ± SD	0.22 ± 0.04	0.24 ± 0.05	0.23 ± 0.07	0.23 ± 0.05		
$PaCO_2$ (mmHg)	Median (IQR)	55.65 (4.85)	54.65 (13.43)	47.45 (6.63)	46.45 (14.18)	0.439	0.889
	Mean ± SD	55.05 ± 4.15	50.55 ± 7.76	48.83 ± 5.47	50.37 ± 10.93		
Bicarbonate (mmol/L)	Median (IQR)	33 (5.1)	31 (6.23)	31.25 (8.05)	31.8 (8.35)	0.302	0.986
	Mean ± SD	33.1 ± 2.51	30.6 ± 4.24	30.79 ± 4.52	30.51± 4.21		
Base excess (mmol/L)	Median (IQR)	7.2 (5.25)	5.25 (4.68)	6.35 (7.25)	6.05 (6.9)	0.365	0.916

	Mean ± SD	7.13 ± 2.6	4.95 ± 3.99	5.48 ± 4.45	4.95 ± 3.61		

Table S4. Effects of dietary supplementation of fish oil and sunflower seed oil on pH, PaO_2, SaO_2, oxygen content, oxygen capacity, alveolar-arterial gradient, $PaCO_2$. Bicarbonate and base excess. LPS = lipopolysaccharide, n = number of rats that survived.

[1]Group B compared with that in group A.

[2]Statistical comparison among groups B, C, and D.

Groups / Variables		A	B	C	D	P^1	P^2
BUN (mg/dL)	Median (IQR)	21.5 (4.05)	106.1 (109.95)	62.65 (104.7)	36.35 (88.65)	**0.008	0.867
	Mean ± SD	21.47 ± 2.31	85.68 ± 52.29	77.13 ± 59.63	59.93 ± 43.19		
CRE (mg/dL)	Median (IQR)	0.2 (0.05)	0.4 (0.7)	0.3 (0.2)	0.2 (0.65)	0.08	0.75
	Mean ± SD	0.22 ± 0.04	0.61 ± 0.59	0.43 ± 0.41	0.48 ± 0.41		
GPT (U/L)	Median (IQR)	23.5 (23)	58 (550.75)	88.5 (324.5)	31.5 (923.25)	0.121	0.98
	Mean ± SD	31.5 ± 19.69	255.75 ± 359.98	328.38 ± 604.6	1092.9 ± 2674.83		
Hemoglobin (g/dL)	Median (IQR)	12.25 (1.93)	13.5 (1.53)	13.4 (1.1)	13.9 (1.25)	*0.044	0.439
	Mean ± SD	12.33 ± 1.06	13.56 ± 0.9	13.48 ± 0.7	13.94 ± 0.77		
Hematocrit (%)	Median (IQR)	38 (6)	42 (4.5)	41.5 (3.75)	43.5 (3.75)	*0.043	0.422
	Mean ± SD	38.33 ± 3.33	42.25 ± 2.76	42 ± 2.2	43.4 ± 2.41		
Lactate (mmol/L)	Median (IQR)	3 (0.9)	3.3 (1.58)	3.1 (2.58)	3.5 (1.43)	0.698	0.872
	Mean ± SD	3 ± 0.62	3.23 ± 1	3.61 ± 1.44	3.46 ± 1.11		
Glucose (mmol/L)	Median (IQR)	155 (40.5)	114 (110.5)	160 (95)	149.5 (80.5)	0.272	0.607
	Mean ± SD	155.6 ± 21.35	124.5 ± 57.74	134.43 ± 49.67	147.8 ± 57.09		
LDH (U/L)	Median (IQR)	243 (1545.25)	481 (921.75)	194.5 (745)	174 (632.75)	0.301	0.711
	Mean ± SD	1072.17 ± 2118.99	588.38 ± 470.58	410.13 ± 442.22	1774.2 ± 4499.06		
Na^+ (mmol/L)	Median (IQR)	141.4 (1.9)	142.85 (3.15)	141.2 (3.05)	142.4 (2.13)	0.22	0.141
	Mean ±	141.47 ±	142.51 ±	140.79 ±	142.39 ±		

	SD	1.49	1.69	1.87	1.87		
K^+ (mmol/L)	Median (IQR)	5.35 (1.48)	4.77 (1.17)	5.08 (1.13)	5.33 (1.02)	0.438	0.213
	Mean ± SD	5.22 ± 0.71	4.87 ± 0.72	5.26 ± 0.72	5.37 ± 0.63		
Ca^{2+} (mmol/L)	Median (IQR)	1.39 (0.06)	1.28 (0.21)	1.27 (0.13)	1.31 (0.14)	0.119	0.554
	Mean ± SD	1.38 ± 0.03	1.29 ± 0.11	1.25 ± 0.08	1.3 ± 0.08		
Mg^{2+} (mmol/L)	Median (IQR)	0.52 (0.1)	0.55 (0.04)	0.51 (0.16)	0.56 (0.1)	0.329	0.764
	Mean ± SD	0.52 ± 0.05	0.55 ± 0.02	0.53 ± 0.1	0.54 ± 0.05		

Table S5. Effects of dietary supplementation of fish oil and sunflower seed oil on the levels of BUN, creatinine, GPT, hemoglobin, hematocrit, lactate, glucose, LDH, Na^+, K^+, Ca^{2+}, and Mg^{2+}. BUN = blood urea nitrogen, GPT = glutamate pyruvate transaminase, LDH = lactate dehydrogenase, LPS = lipopolysaccharide, n = number of rats that survived.

[1]Group B compared with that in group A.

[2]Statistical comparison among groups B, C, and D.

1.2 Supplementary Figures

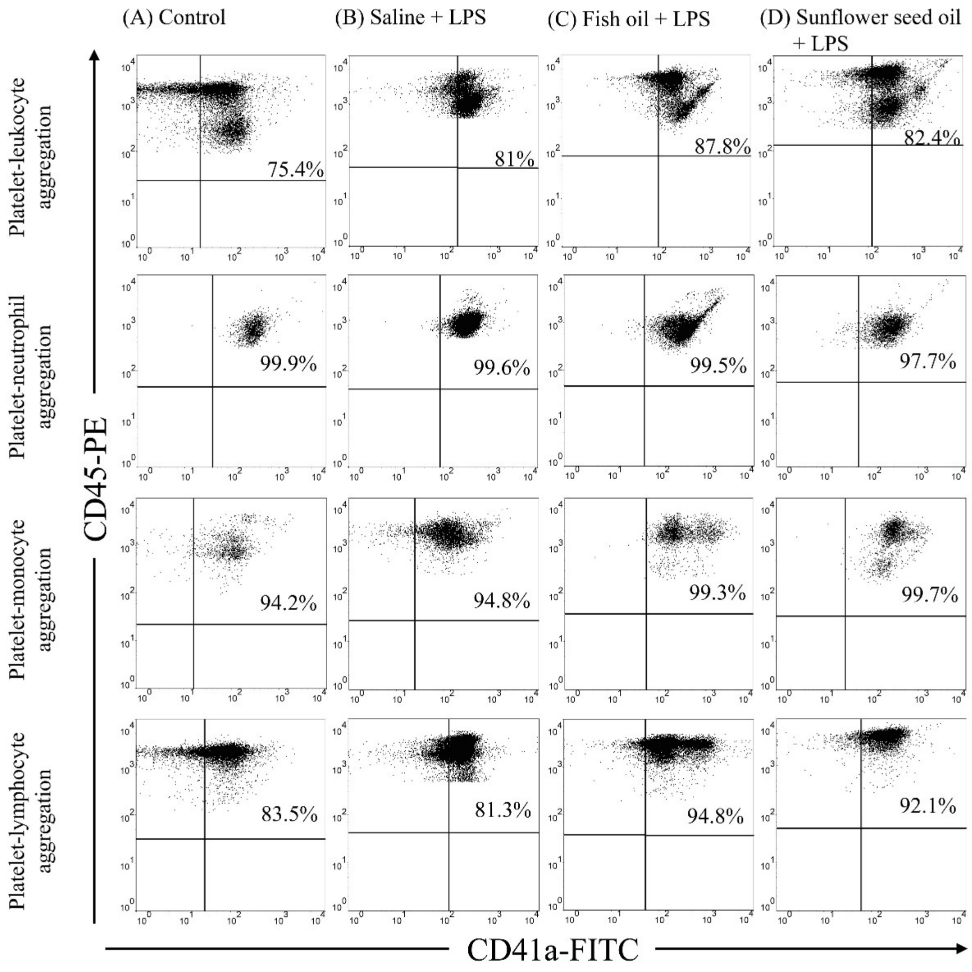

Figure S1. Flow cytometric analysis of platelet-leukocyte aggregation. Platelet-leukocyte aggregation (including neutrophil, monocyte, and lymphocyte) were identified by anti-CD45 antibody (PE; y-axis) and positive labeling with a platelet-specific monoclonal antibody (CD41a-FITC; x-axis). Dot plot of the fluorescence of (A) control group, (B) saline + LPS group, (C) fish oil + LPS group, and (D) sunflower seed oil + LPS group; the percentage of particles positive for both anti-CD45-PE and anti-CD41a-FITC represent the percentage of leukocyte aggregated with platelets. LPS = lipopolysaccharide.

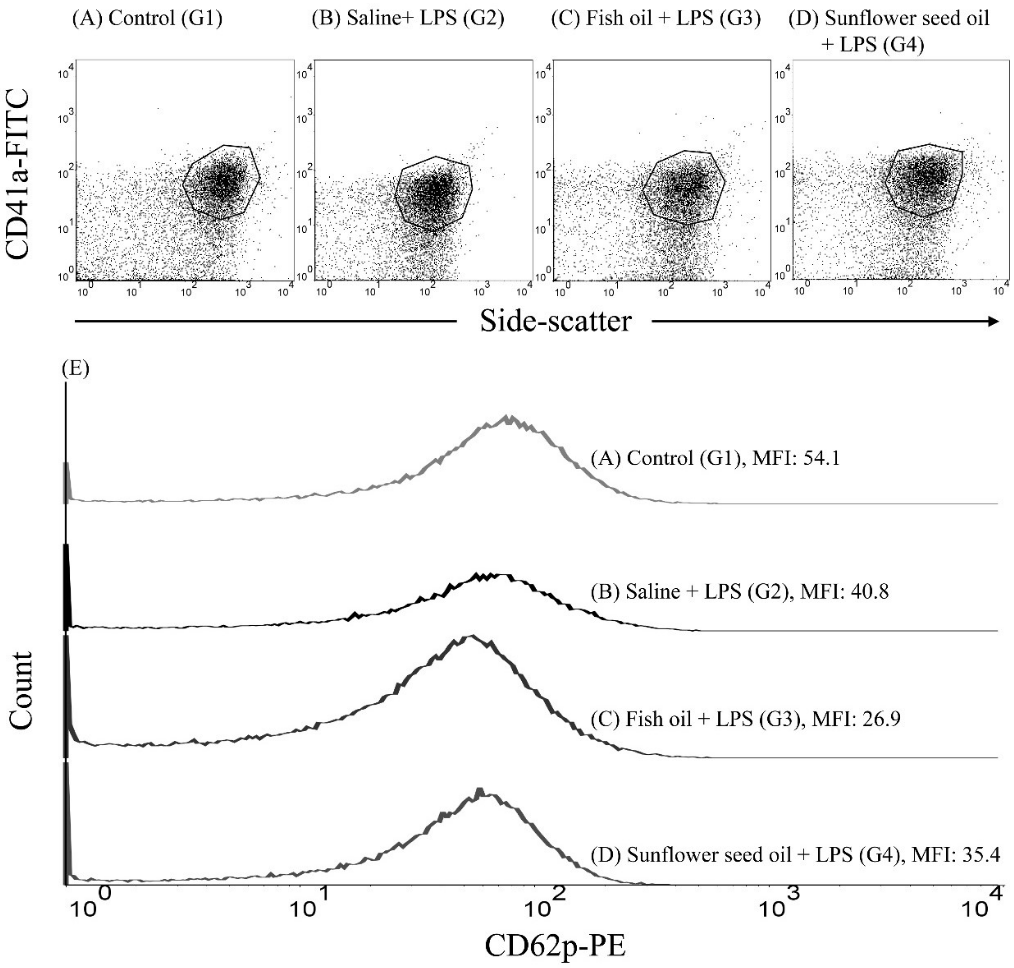

Figure S2. Flow cytometric analysis of platelet P-selectin expression. Individual platelets were identified by their characteristic side-scatter properties (granularity; x-axis) and positive labeling with a platelet-specific monoclonal antibody (CD41a-FITC; y-axis). Dot plot of the fluorescence of (A) control group, (B) saline + LPS group, (C) fish oil + LPS group, and (D) sunflower seed oil + LPS group. (E) Overlay of the histograms of four groups; fluorescent events represent the total number of platelets with P-selectin expression. LPS = lipopolysaccharide.

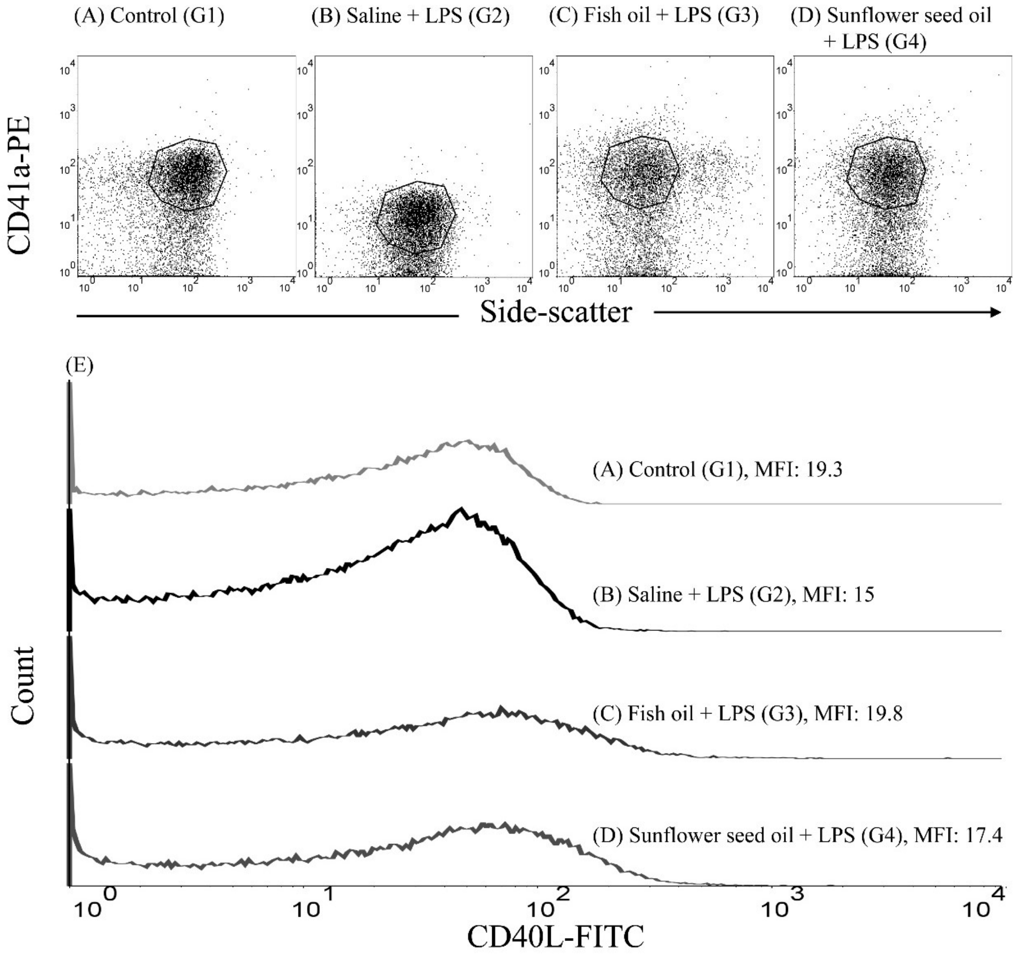

Figure S3. Flow cytometric analysis of platelet CD40L expression. Individual platelets were identified by their characteristic side-scatter properties (granularity; x-axis) and positive labeling with a platelet-specific monoclonal antibody (CD41a-PE; y-axis). Dot plot of the fluorescence of (A) control group, (B) saline + LPS group, (C) fish oil + LPS group, and (D) sunflower seed oil + LPS group. (E) Overlay of the histograms of four groups; fluorescent events represent the total number of platelets with CD40L expression. LPS = lipopolysaccharide.

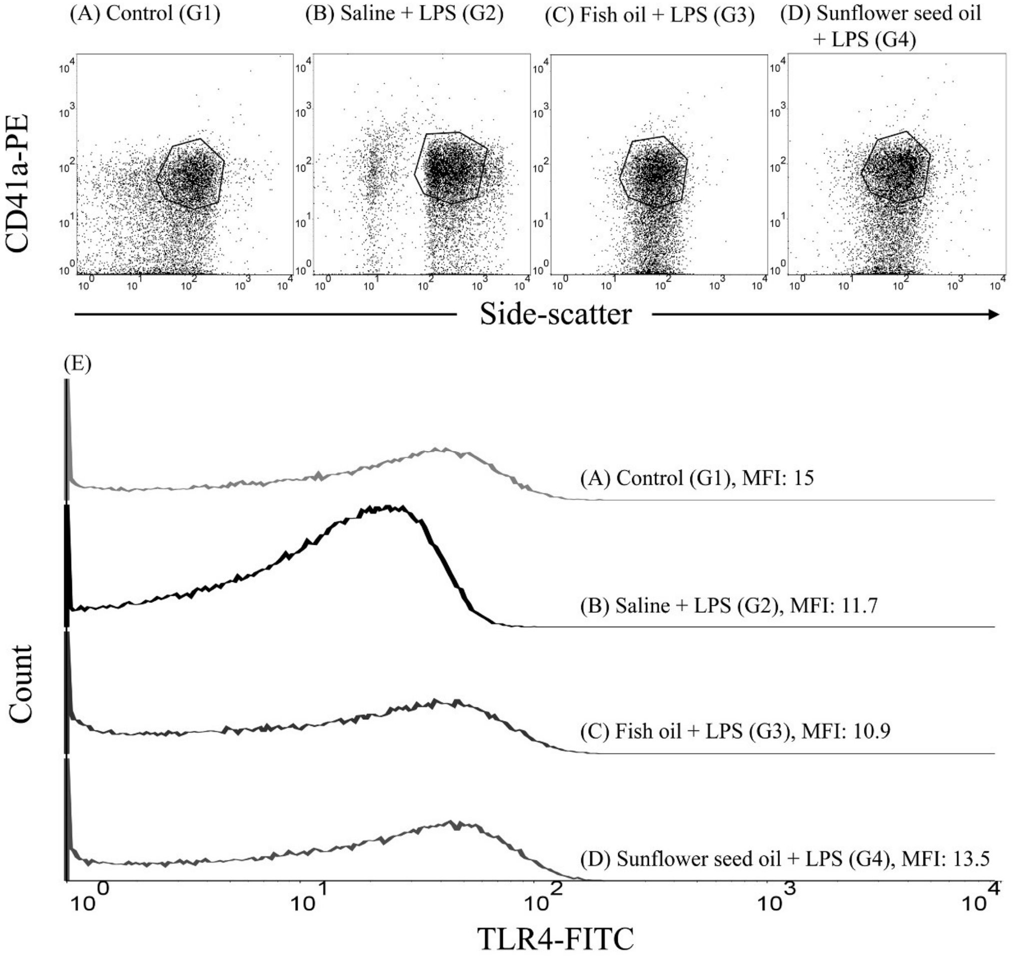

Figure S4. Flow cytometric analysis of platelet TLR4 expression. Individual platelets were identified by their characteristic side-scatter properties (granularity; x-axis) and positive labeling with a platelet-specific monoclonal antibody (CD41a-PE; y-axis). Dot plot of the fluorescence of (A) control group, (B) saline + LPS group, (C) fish oil + LPS group, and (D) sunflower seed oil + LPS group. (E) Overlay of the histograms of four groups; fluorescent events represent the total number of platelets with TLR4 expression. LPS = lipopolysaccharide.

References:

1. Brealey D, Karyampudi S, Jacques TS, Novelli M, Stidwill R, Taylor V, et al. Mitochondrial dysfunction in a long-term rodent model of sepsis and organ failure. *Am J Physiol Regul Integr Comp Physiol* (2004) 286(3):R491-7. Epub 2003/11/08. doi: 10.1152/ajpregu.00432.2003. PubMed PMID: 14604843.
